# Retraction: MiRNA-574-3p inhibits cell progression by directly targeting CCND2 in colorectal cancer

**DOI:** 10.1042/BSR-2019-0976_RET

**Published:** 2021-08-24

**Keywords:** colorectal cancer (CRC), Cyclin D2 (CCND2), microRNA-574-3p, migration, proliferation

This Retraction follows an Expression of Concern relating to this article previously published by Portland Press.

This article is being retracted from *Bioscience Reports* at the request of the Editor-in-Chief and the Editorial Board following receipt of a notification from a reader, alerting the authors to duplicated sections in Figures 3A, 3B and 4A.

The authors have been contacted in regards to the Retraction, and have not responded to the Journal's queries and the concerns raised. Given the extent of the issues raised, the Editorial Board stand by the decision to retract the article.

